# Comparative Analysis of Dynamic Cell Viability, Migration and Invasion Assessments by Novel Real-Time Technology and Classic Endpoint Assays

**DOI:** 10.1371/journal.pone.0046536

**Published:** 2012-10-19

**Authors:** Ridha Limame, An Wouters, Bea Pauwels, Erik Fransen, Marc Peeters, Filip Lardon, Olivier De Wever, Patrick Pauwels

**Affiliations:** 1 Center for Oncological Research (CORE), University of Antwerp, Antwerp, Belgium; 2 StatUA Center for Statistics, University of Antwerp, Antwerp, Belgium; 3 Department of Oncology, Antwerp University Hospital, Edegem (Antwerp), Belgium; 4 Laboratory of Experimental Cancer Research, Department of Radiotherapy and Nuclear Medicine, Ghent University Hospital, Ghent, Belgium; 5 Laboratory of Pathology, Antwerp University Hospital, Edegem (Antwerp), Belgium; Wayne State University School of Medicine, United States of America

## Abstract

**Background:**

Cell viability and motility comprise ubiquitous mechanisms involved in a variety of (patho)biological processes including cancer. We report a technical comparative analysis of the novel impedance-based *xCELLigence* Real-Time Cell Analysis detection platform, with conventional label-based endpoint methods, hereby indicating performance characteristics and correlating dynamic observations of cell proliferation, cytotoxicity, migration and invasion on cancer cells in highly standardized experimental conditions.

**Methodology/Principal Findings:**

Dynamic high-resolution assessments of proliferation, cytotoxicity and migration were performed using *xCELLigence* technology on the MDA-MB-231 (breast cancer) and A549 (lung cancer) cell lines. Proliferation kinetics were compared with the Sulforhodamine B (SRB) assay in a series of four cell concentrations, yielding fair to good correlations (Spearman's Rho 0.688 to 0.964). Cytotoxic action by paclitaxel (0–100 nM) correlated well with SRB (Rho>0.95) with similar IC_50_ values. Reference cell migration experiments were performed using Transwell plates and correlated by pixel area calculation of crystal violet-stained membranes (Rho 0.90) and optical density (OD) measurement of extracted dye (Rho>0.95). Invasion was observed on MDA-MB-231 cells alone using Matrigel-coated Transwells as standard reference method and correlated by OD reading for two Matrigel densities (Rho>0.95). Variance component analysis revealed increased variances associated with impedance-based detection of migration and invasion, potentially caused by the sensitive nature of this method.

**Conclusions/Significance:**

The *xCELLigence* RTCA technology provides an accurate platform for non-invasive detection of cell viability and motility. The strong correlations with conventional methods imply a similar observation of cell behavior and interchangeability with other systems, illustrated by the highly correlating kinetic invasion profiles on different platforms applying only adapted matrix surface densities. The increased sensitivity however implies standardized experimental conditions to minimize technical-induced variance.

## Introduction

Among the most fundamental hallmarks of cancer are loss of pre-existing tissue architecture by sustained proliferation and extracellular matrix infiltration of cancer cells. Cancer cells may sustain proliferative signaling in an autocrine or paracrine fashion by producing growth factors themselves, by overexpression of growth factor receptors or by a constitutive activation of downstream signaling components [1]. Monitoring of cell proliferation and cell viability is critical in biomedical research, in order to understand the pathways regulating proliferation and viability, and to develop agents that modulate these processes. The sulforhodamine B (SRB) test is a high throughput and reproducible colorimetric assay, based on the binding of SRB to protein basic amino acid residues, providing a sensitive index of cellular protein content that is linear over a cell density range [2].

Matrix penetration necessitates activation of the cellular motility apparatus and can occur by either individual cells or cell strands, sheets or clusters [3]. A phenomenon predominantly involved in this process is chemotaxis, whereby cell movement is directed along an extracellular chemical gradient of secreted factors in the microenvironment [4]. Already in the early stages of embryogenesis, formation of complex tissues and organs is orchestrated by fine-tuned chemotactic migration of cell chains. In malignant processes however, cancer cells tend to adopt similar, if not identical mechanisms to metastasize to distant organ sites [5]. Several well-established experimental approaches are available to study cell migration and chemotaxis *in vitro* (reviewed in [6]). The Transmembrane/*Boyden chamber* assay is based on a chemotactic-driven cell transit through a filter [7]. An important feature of the endpoint in this experimental set-up is that cells need to exhibit active migratory behavior to end up at the other side of the membrane.

**Figure 1:**
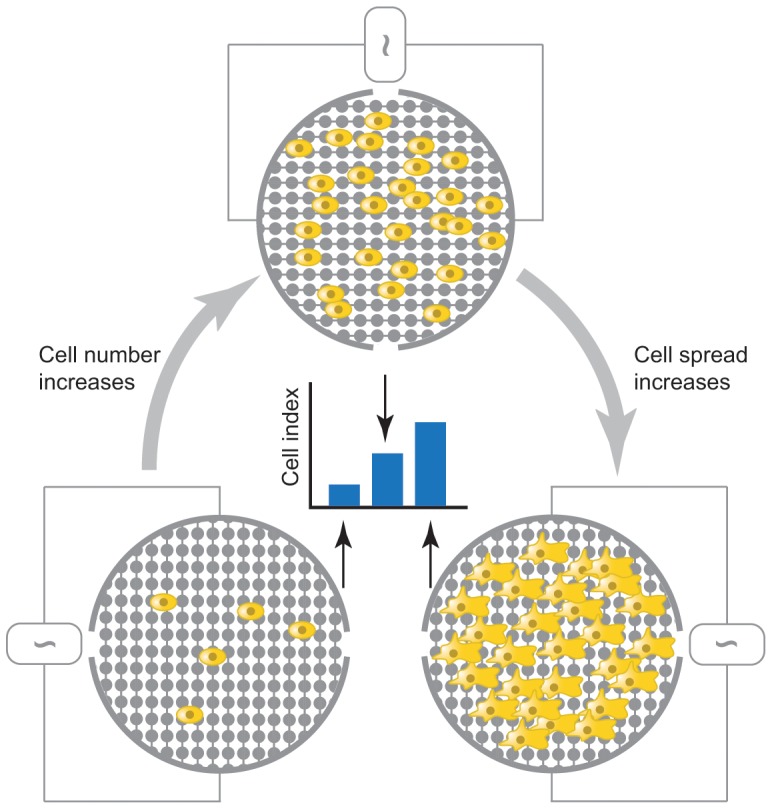
*xCELLigence* RTCA: impedance-based detection of cell viability and motility. Interdigitated gold microelectrodes on the well bottom (viability – E-plate) or on the bottom side of a filter membrane (motility – CIM-plate 16) detect impedance changes, caused by the presence of cells and expressed as a Cell Index. This detection method is proportional to both cell number (*left* and *above*) and morphology as increased cell spreading is reflected by a higher Cell Index value (*right*). When starting an experiment, a baseline Cell Index value is recorded in medium only before cell addition.

The *xCELLigence RTCA* technology (Roche Applied Science) has emerged as an alternative non-invasive and label-free approach to assess cellular proliferation, migration and invasion in real time on a cell culture level [8]. This system makes use of impedance detection for continuous monitoring of cell viability, migration and invasion (reviewed in [9]) (Fig. 1).

Here we report data of *in vitro* assessment of four cellular processes (proliferation, cytotoxicity, migration and invasion) on the MDA-MB-231 and A549 cancer cell lines using *xCELLigence* RTCA DP (Roche Applied Science) in comparison with data resulting from parallel experiments applying a previously existing and well-established measuring method (to be considered as a “gold standard” method) for each process. Both these cell lines are extensively characterized and used as models representing two different highly incidental tumor types (breast cancer, lung cancer). Furthermore, these cell lines show a strong degree of motility in the wild-type state, thus providing useful examples for the distinction between chemotactic and random motility.

Importantly, all of the comparative techniques are traditional label-based endpoint assays that have been selected due to their widespread application within the scientific community and similarity in working principle with *xCELLigence*. Although they have been slightly modified to match with the *xCELLigence* setup and its ability to acquire time-dependent kinetics of cultured cell behavior, the fundamental handling and detection principles of each classic assay have been maintained. Cell proliferation and cytotoxicity testing has been performed using the SRB assay [10], with the microtubule stabilizer paclitaxel as cytotoxic agent in the latter experiments. Being widely used for the treatment of a variety of tumor types, inhibitory effects of this anti-mitotic compound on cell proliferation have been described extensively. Furthermore, previous reports on the use of the *xCELLigence* device included paclitaxel as a reference compound in their studies [8], [11], making this a suitable agent for this study. Cell migration and invasion experiments were performed using conventional Transwell plates and quantified by both pixel area calculation of stained membranes and optical density reading of solubilized dye. For the first time, results from “tried-and-tested” assay setups are confronted with parallel data recorded using a novel, commercially available technology, providing an objective technical comparison of dynamic observations on cultured cells in highly standardized experimental conditions.

## Results

### Proliferation

The dynamic assessment of proliferation kinetics was modeled by performing SRB testing on both MDA-MB-231 and A549 cells. Growth curve studies were performed over a ten-day period and proliferation curves were established for four different plating densities. To correct for seeding area differences between SRB-experiments and *xCELLigence*, cell seeding densities were synchronized between both techniques (100, 500, 1000 and 2000 cells/cm^2^). Corresponding experiments on the *xCELLigence* system were performed in duplicates and the resulting high-resolution data were extrapolated to the matching data points of the counterpart method as described in the Materials and Methods section.

For MDA-MB-231 cells, cell doubling time was 27.78±5.14 hours and 29.92±2.85 hours measured with the SRB assay and the xCELLigence system respectively. Similarly, for A549 cells, doubling time was 27.93±1.75 hours and 29.18±1.87 hours measured with the SRB assay and the xCELLigence system respectively.

**Figure 2:**
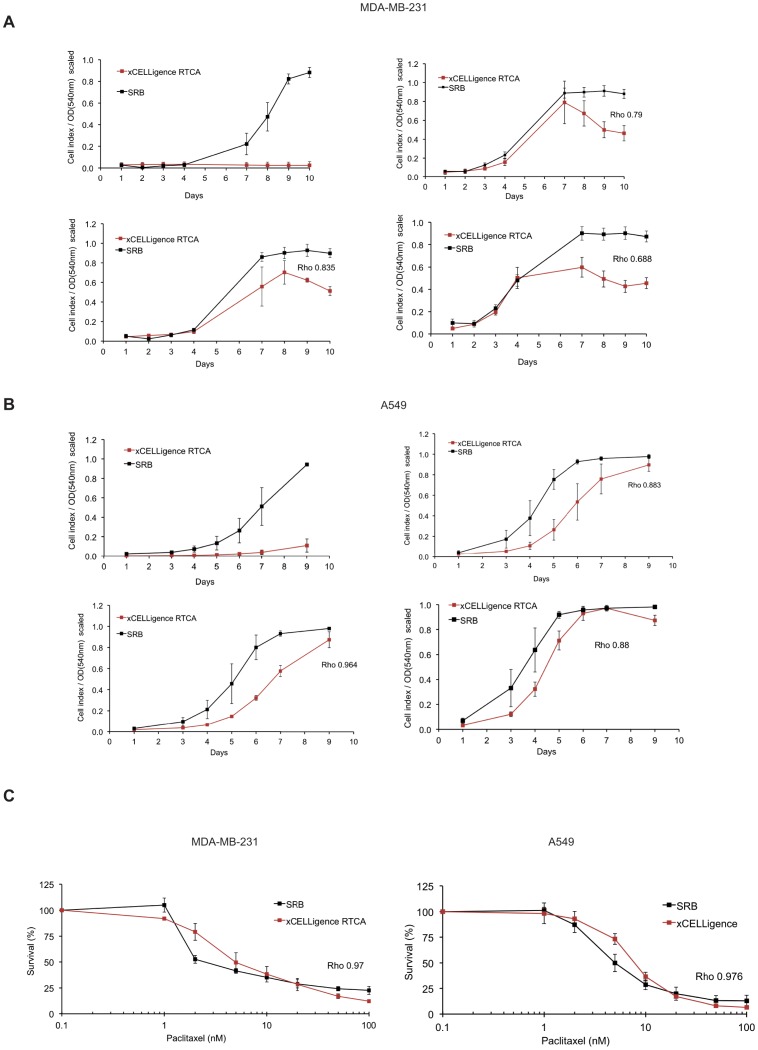
Time-dependent proliferation and cytotoxicity profiles of MDA-MB-231 and A549. A. Proliferation curves of MDA-MB-231 cells as generated by *xCELLigence* RTCA (*red*) and SRB (*black*) for different seeding densities of 100 (*top left*), 500 (*bottom left*), 1000 (*top right*) and 2000 cells/cm^2^ (*bottom right*) during a ten-day incubation. B. Same as (A) for A549 cells. All graphs represent results from three independent experiments ± SD. C. Cytotoxicity profiles relating to 72 hours of exposure to paclitaxel (0–100 nM). Cells were allowed to attach and propagate during 24 hours prior to start of treatment. Toxicity data from *xCELLigence* RTCA were derived from normalized plots. All graphs represent results from three independent experiments ± SD.

Spearman's Rho (ρ) correlations were calculated on global average results that had been normalized to the highest value in the data set per method (SRB or *xCELLigence*) to eliminate units of measurement (Fig. 2A, B, shown as “scaled”). Both MDA-MB-231 and A549 cells revealed fair to good correlation rates for all applied seeding densities, noting however that proliferation did not set off at the lowest cell seeding density (100 cells/cm^2^) of MDA-MB-231 on RTCA (Fig. 2A). A549 cells showed only minimal proliferative activity at this density as well (Fig. 2B). Correlations observed between SRB-based and impedance-based quantitation reached higher values at the medium cell seeding densities of 500 cells/cm^2^ and 1000 cells/cm^2^ for both cell lines tested (Spearman's ρ = 0.835 resp. 0.790 for MDA-MB-231 and ρ = 0.964 resp. 0.883 for A549). Altogether, correlation values ranged from 0.880 to 0.964 for A549 and 0.688 to 0.835 for MDA-MB-231. Variance component analysis on proliferation data of A549 cells resulting from both techniques indicated smaller intra- and inter-experimental variances on *xCELLigence* when compared to SRB. Conversely, detection of proliferation kinetics of MDA-MB-231 cells resulted in a higher degree of (intra- and interexperimental) variance when performed with the *xCELLigence* system (quantified as σ_w_ and σ_b_ in Table S1).

### Cytotoxicity

Compound cytotoxicity was assessed after exposure of MDA-MB-231 and A549 cells to a concentration range of paclitaxel (0–100 nM for 72 hours). In both cell lines, a similar toxic response was detected by both SRB and *xCELLigence*, with comparable IC_50_ values of 4.78±0.90 nM and 6.44±1.90 nM respectively (t-test, p = 0.244) in MDA-MB-231 cells. The normalized toxic response correlated highly between SRB-based and *xCELLigence*-based detection for both cell lines (Spearman's ρ = 0.970 and 0.976 for MDA-MB-231 and A549 resp.) (Fig. 2C).

### Cell migration

**Figure 3:**
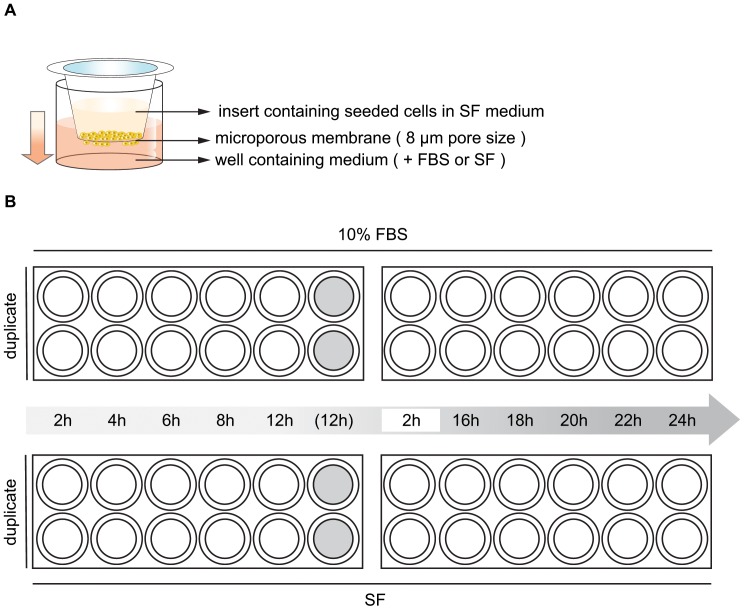
Conventional Transwell design for detection of time-dependent cell migration. A. A Transwell setup consists of an upper chamber (*insert*) that is placed onto a lower chamber (*well*). The insert contains a microporous membrane (8 µm pores) allowing passage of tumor cells. After a period of serum starvation a serum-free cell suspension is seeded in the insert and exposed to medium containing potential chemoattractants (by default: medium+FBS). During incubation at 37°C and 5% CO_2_, cells migrate toward the bottom side of the membrane. B. Experimental design to assess time-dependent migratory behavior of cultured cells. Both migration toward FBS-containing medium and baseline migration (toward SF medium, no chemoattraction) as a negative control were included. Two times two 24-well Transwell plates were used to examine migration to FBS (positive control – *top row*) and baseline migration (negative control – *bottom row*). At ten time points during a 24-hour incubation period inserts were fixed and stained in duplicate. Two inserts containing cell-free media (grey fill) have been included throughout the experiment and fixed and stained after 12 hours incubation to assess background absorption in optical density (OD) measurements. In addition, to exclude influence of inter-plate variability on observed migration rates, each plate contained duplicate two-hour control inserts.

**Figure 4:**
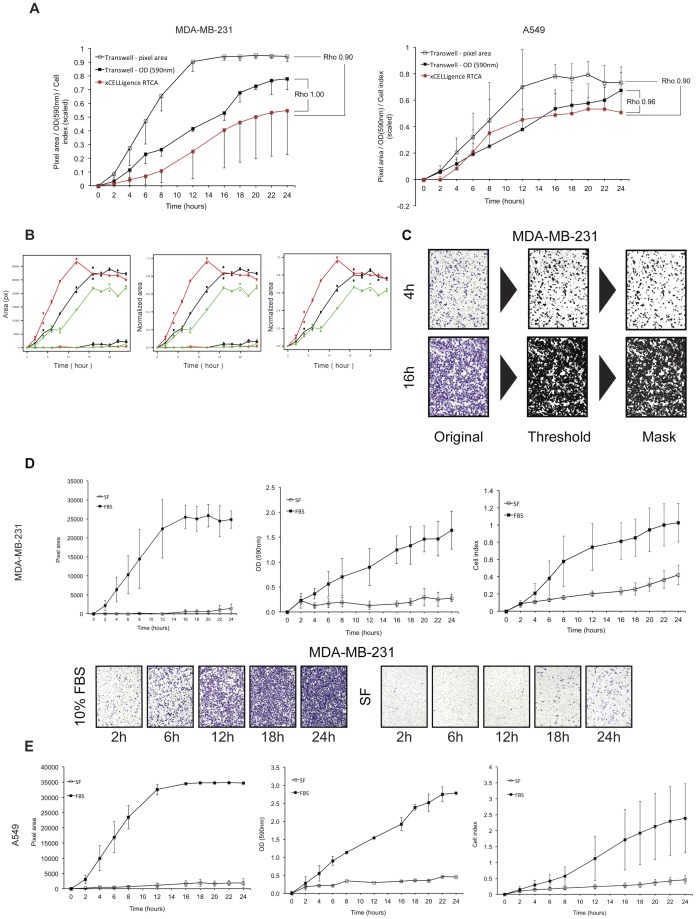
Time-dependent migratory pattern of MDA-MB-231 and A549. A. MDA-MB-231 (*left*) and A549 (*right*) cell migration profiles, detected by Transwell experiments (*black*) and *xCELLigence* (*red*). Graphs represent scaled signals (0–1) of net chemoattraction after subtraction of the random migration signal (*empty squares* in panel A, B), with associated Spearman's Rho values. All results originate from three independent duplicate experiments ± SD. B. Normalization procedure of migration patterns. Raw data (*left panel*) were normalized to a (0–1) scale (*middle panel*) through division of all data by the maximum value obtained in three independent experiments. Subsequently, random migration (SF) signals (*triangle markers*) were subtracted from the positive (FBS) control counterparts (*circle markers*) per experiment to obtain a pure chemotactic signal (*right panel*). Example shown is the migratory pattern of MDA-MB-231 cells estimated by pixel area calculation in three experiments (exp 1 - *red*, exp 2 - *green*, exp 3 - *black*). Triangle and circle markers represent negative (SF) and positive (FBS) control data respectively. C. ImageJ-based picture processing. Original pictures were color thresholded to obtain a binary image displaying cellular content as saturated black areas on a white background. Thresholded images were masked to exclude non-cellular particles from the final area calculation. Pictures shown are migrated MDA-MB-231 cells after four hours (*top row*) and 16 hours (*bottom row*) of incubation. D. Migratory behavior of MDA-MB-231 cells toward medium+FBS (positive control – *filled squares*) and background migration (*empty squares*) as detected by conventional Transwell experiments at ten time points spread over 24 hours of incubation. Area calculation (*left*) of stained cells and optical density (OD – *middle*) were compared to the *xCELLigence* migration pattern, reconstructed from the original high-resolution plot by extrapolating data from the corresponding time points (*right*). All results represent original data from three independent duplicate experiments ± SD. Picture string (obj. 2.5×) shows migratory status of MDA-MB-231 cells, stained as described, at five different stages during 24 hours of incubation. E. Same as (D) for A549 cells.

Conventional Transwell plates have been organized in a sequential setup to perform dynamic observations of cancer cell migration in a time-dependent manner, yielding a series of data similar to the *xCELLigence* system (Fig. 3A, B). Both chemotactic migration to medium containing 10% FBS and random migration with SF medium on either side of the membrane have been considered. High correlation values for both cell lines were obtained when comparing area calculation and OD with *xCELLigence* Cell Index (CI) (Fig. 4A). Importantly, it must be noted that correlation coefficients were calculated for overall mean values resulting from three independent experiments, each performed in duplicates for all time points. As described in the Materials and Methods section, all raw data obtained were normalized to the single maximal value over three experiments per quantitation method (CI, pixel area or OD) to eliminate units of measurement. Subsequently, values from random (SF) migration were subtracted from chemotactic (FBS) migration per time point to generate signals of net chemoattraction (Fig. 4B). Pixel area calculations, averaged over three fields per insert membrane (Fig. 4C), correlated with *xCELLigence* CI measurements for both MDA-MB-231 and A549 cells (Spearman's ρ = 0.90 for both cell lines). However, OD measurements showed even stronger correlations with *xCELLigence* data (Spearman's ρ = 0.96 and 1.00 for MDA-MB-231 and A549 resp.). Area calculation correlated with OD measurements in a similar fashion as with *xCELLigence* for both MDA-MB-231 and A549 (Spearman's ρ = 0.89 and ρ = 0.90 resp.) (Fig. 4A).

At later time points of the experiments, *xCELLigence* generated larger variances between intra-experimental replicates when compared to area calculations or OD values derived from classic Transwells (Table S1). Briefly, variance between replicates within one experiment increased over time, creating a funnel shaped time-dependent pattern (Fig. 4D, E). Variance component analysis indeed revealed an increase of intra-experimental variance on *xCELLigence* data in comparison with Transwell data. However, early (<10 h) pixel area values showed similar degrees of variance. Additional experiments using an identical setup yielded similar results (results not shown).

**Figure 5:**
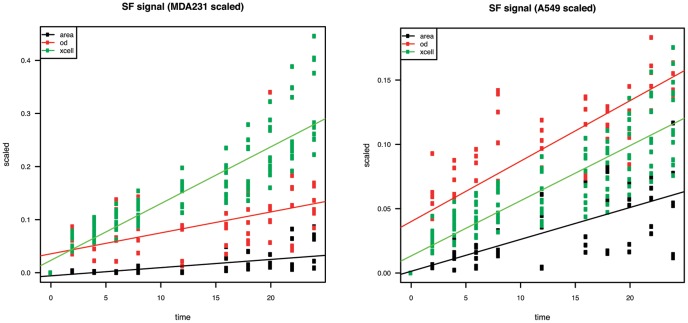
Time-dependent random migration profile of MDA-MB-231 and A549. Comparison of random migration signals (negative control – SF) between three quantitation methods: pixel area calculation – *black*, OD - *red*, xCELLigence - *green*. A likelihood ratio test revealed a significant difference in slope between area calculation and OD (p<0.001) and area calculation and *xCELLigence* (p<0.001) for both cell lines and OD and *xCELLigence* (p<0.001) for MDA-MB-231 only. OD and *xCELLigence* slopes did not differ significantly (p = 0.22) for A549 cells.

A significant difference was observed between background (serum-free, SF) signals generated by the three methodologies of cell migration quantitation. These signals derived from random movement of cells without exposure to any chemoattractant and, serving as a negative control, showed a different pattern when generated by *xCELLigence* or by both quantitation techniques using Transwells (Fig. 5). Paired comparisons between time-dependent tracking of background migration were performed between all techniques using a likelihood ratio test and revealed a significant difference between *xCELLigence* and pixel area calculation (p<0.001) for both MDA-MB-231 and A549. OD measurements differed significantly from *xCELLigence* (p<0.001) for MDA-MB-231 cells, but showed a similar pattern when compared with *xCELLigence* data generated from A549 cells (p = 0.22).

### Cell invasion

**Figure 6:**
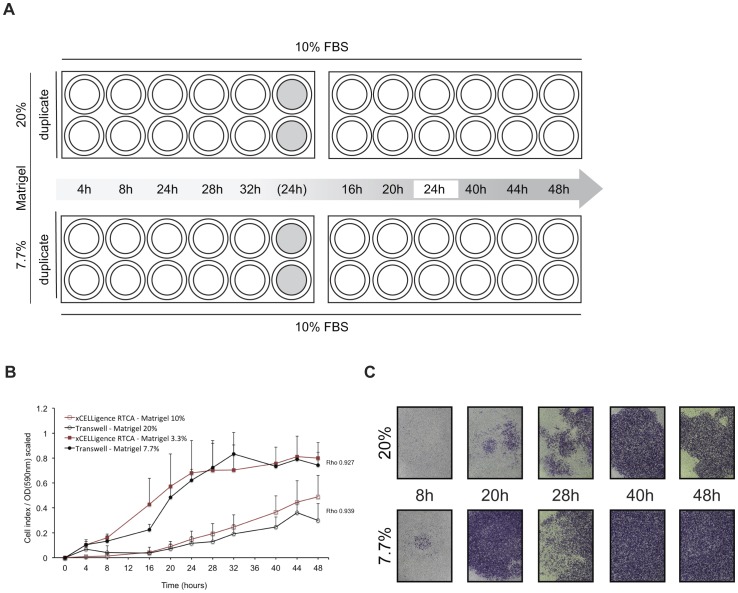
Time-dependent invasion profile of MDA-MB-231. A. Experimental design to quantify MDA-MB-231 Matrigel invasion. Two times two 24-well Transwell plates were used to examine invasion to FBS through a 20% (v/v) (*top row*) and 7.7% (v/v) Matrigel layer (*bottom row*) after 24 hours of serum starvation. At ten time points during a 48-hour incubation period inserts were fixed and stained in duplicate. Two inserts containing cell-free media (grey fill) have been included throughout the experiment and fixed and stained after 24 hours incubation to assess background absorption in optical density (OD) measurements. In addition, to exclude influence of inter-plate variability on observed migration rates, each plate contained duplicate 24-hour control inserts. B. MDA-MB-231 dynamic cell invasion profiles, generated by Transwell experiments (*black*) and *xCELLigence* (*red*). Graphs represent normalized signals (scaled values 0–1) of invasion through 20% (*open circles*), 10% (*open squares*), 7.7% (*filled circles*) and 3.3% (*filled squares*) to medium+10% FBS with associated Spearman's rank correlation coefficients (Rho). All results are from three independent duplicate experiments with SD. C. Sequential pictures showing invasive MDA-MB-231 cells at the indicated time points during a 48-hour incubation on Transwells coated with 20% (*top row*) and 7.7% Matrigel (*bottom row*). Pictures (obj. 2.5×) show cells fixed and stained in 20% methanol/0.1% crystal violet.

Impedance-based detection of MDA-MB-231 cell invasion was compared with results derived from a Transwell system as applied for migration experiments, with Matrigel as extracellular matrix component added on top of the microporous membranes (Fig. 6A). It was found that Matrigel dilutions of 10% (v/v, SF medium) on *xCELLigence* yielded high correlations when compared to a dilution of 20% on Transwells in dynamic invasion profile recording during a 48-hour incubation (Spearman's ρ = 0.939). Similarly, invasion through a Matrigel dilution of 3.3% on *xCELLigence* correlated highly with a dilution of 7.7% on Transwell plates (Spearman's ρ = 0.927) (Fig. 6B, C). Variance component analysis of Transwell and *xCELLigence* data revealed slightly increased degrees of intra-experimental variance in the latter. The variance between independent experiments was increased for *xCELLigence* in comparison with Transwell assays (Table S1). All Matrigel dilutions have been synchronized regarding seeding surface area for both systems and theoretical calculations for correlating Matrigel dilutions are shown in Table 1.

**Table 1:** Matrigel surface densities corresponding with degree of dilution for a fixed volume of 20 µL.

Matrigel density		
xCELLigence RTCA		Transwell
%	µg/cm^2^ *	%
10.0	189.50	23.1
3.3	63.17	7.7

* Matrigel (Basement Membrane Matrix, growth factor reduced, BD Biosciences) delivered as a ±13.55 µg/µL stock.

## Discussion

*xCELLigence* technology measures impedance changes in a meshwork of interdigitated gold microelectrodes located at the well bottom (E-plate) or at the bottom side of a microporous membrane (CIM16-plate). These changes are caused by the gradual increase of electrode surface occupation by (proliferated/migrated/invaded) cells during the course of time and thus can provide an index of cell viability, migration and invasion. This method of quantitation is directly proportional to cellular morphology, spreading, ruffling and adhesion quality as well as cell number [8], [9] (Fig. 1). Cell proliferation and paclitaxel cytotoxicity kinetics, as assessed by the *xCELLigence* platform, were compared with an SRB-based approach, showing good correlations for both cell lines tested, implying that both methods detect similar process kinetics when performed in standardized conditions. However, correlations were generally stronger for the A549 than for the MDA-MB-231 cell line, which may indicate a possible cell type-dependent cause. MDA-MB-231 cells show a heterogeneous morphotype with round and spread cells, which may differentially influence impedance based measurements or crystal violet uptake. Paclitaxel was chosen as it is widely used as treatment for a variety of tumor types and previous reports on the use of the *xCELLigence* system as a tool for cytotoxicity screening included paclitaxel as a reference compound [8], [11]. Nevertheless, it must be underlined that the highly correlative nature of cytotoxic kinetics as detected by both techniques for paclitaxel may not apply for certain other compounds. Indeed, the *xCELLigence* RTCA device has been reported to generate compound-specific kinetic profiles on cultured cells, hereby demonstrating associations with the respective mechanisms of action [11].

To quantify cell migration through conventional setups, detection by crystal violet was selected, followed by pixel area and OD quantitation, as these methods are widespread within the scientific community and also take morphologic features into account. Both area calculation and OD measurement correlated highly with cell migration, as detected by *xCELLigence*, confirming that the observed kinetic cell behavior is strongly similar, provided that equal cell seeding densities are applied. The closest associations were found between OD measurements and RTCA CI, generating nearly identical migration patterns. OD values were determined on cell lysates with extracted crystal violet stain, derived from entire Transwell membranes, and thus provide a more reliable quantitation when compared with pixel area calculation, which was based on averaging three microscopic fields per Transwell membrane. This indicates that impedance-based measurements have smaller limits of detection, resulting in highly reliable migration estimates. Analysis of background signals, resulting from random migration in a serum-free environment (negative control), revealed a significant difference in signal detection between *xCELLigence* and both classic techniques (Fig. 5). Weak signals resulting from limited baseline cell migration were indeed more accurately detected by the RTCA platform, which implies a higher sensitivity, compared to classic detection methods. OD measurements correlated more closely with *xCELLigence* data in all experiments and did not show a significant difference when background signals were compared with *xCELLigence* for the A549 cell line, suggesting a smaller detection limit and thus a more accurate method of quantifying cell migration when using Transwells. Additionally, a smaller limit of detection can explain the observed time-dependent increase of variability between intra-experimental replicates during the course of an experiment. Data derived from cell culture-based experiments are subject to inter- as well as intra-individual variation regarding cell counting, pipetting, preparation of chemotactic factors and general cell culture handling. As a consequence, small handling differences during the preparation of an experiment can result in signal differences between technical replicates and this will be reflected in variations increasing with time within one experiment when compared to area calculation or OD measurement, that do not detect this variability to this extent. This is also illustrated by cell culture-based invasion experiments, as the uniform application of a Matrigel layer implies the introduction of an important added variable. Consequent Matrigel thawing and handling on ice, using only cooled consumables, and hands-on coating experience can contribute to homogenous gelification and thus to enhanced assay reproducibility. Comparative invasion results have shown correlation between *xCELLigence* and Transwells when similar cell seeding densities were applied and, more importantly, when the amount of Matrigel per square area unit is synchronized. Diluting the matrix barrier, and thus changing the degree of matrix fenestration, gives rise to similar invasion rates on both setups with different seeding areas, although equal volumes of Matrigel have been applied.

In conclusion, the real-time label-free *xCELLigence* system provides a suitable and accurate platform for high-throughput kinetic screenings and for determination of cell motility dynamics. In contrast with classic endpoint assays, the impedance-based detection method is generally less labor-intensive, provides kinetic information on the studied processes and does not affect cell viability, potentially generating further experimentation possibilities. Moreover, the correlating observations as performed with conventional approaches make methods interchangeable to perform functional studies when larger cell populations of interest are needed. However although impedance measurement provides a sensitive cell-based detection method, it should be applied as a complementary tool to further functional confirmation.

Importantly, this is the first study illustrating the highly correlative nature of invasion kinetics detected by two different setups applying synchronized matrix densities. The increased sensitivity, however, necessitates standardized experimental conditions and user experience, to minimize variance increments on the *xCELLigence* system.

## Materials and Methods

### Cell culture

Two malignant cell lines (A549, lung adenocarcinoma; MDA-MB-231, breast adenocarcinoma), obtained from the American Type Culture Collection (ATCC, Manassas, VA, USA) (http://www.lgcstandards-atcc.org), were cultured in DMEM and RPMI1640 respectively, each supplemented with 10% fetal bovine serum (FBS), 1% penicillin/streptomycin, 1% L-glutamine and additionally, 1% sodium pyruvate was added to RPMI1640 only. All cell culture reagents were purchased from Invitrogen NV/SA (Merelbeke, Belgium). For proliferation and cytotoxicity experiments, normal growth medium containing FBS was used. Cell lines were maintained at 37°C and 5% CO_2_/95% air in a humidified incubator and confirmed free of mycoplasma infection through regular testing (*MycoAlert® Mycoplasma Detection Kit*, Lonza, Belgium). All cell lines have been validated in-house by short tandem repeat (STR) profiling using the Cell ID™ System (Promega, Madison, WI, USA) according to the manufacturer's instructions. The obtained STR profiles were matched with reference ATCC DNA fingerprints (www.lgcstandards-atcc.org) and with the Cell Line Integrated Molecular Authentication (CLIMA) database (http://bioinformatics.istge.it/clima) [12] to authenticate cell line identity.

### *xCELLigence Real-Time Cell Analysis (RTCA): proliferation and cytotoxicity*

Experiments were carried out using the *xCELLigence* RTCA DP instrument (Roche Diagnostics GmbH, Mannheim, Germany) which was placed in a humidified incubator at 37°C and 5% CO_2_.

Cell proliferation and cytotoxicity experiments were performed using modified 16-well plates (E-plate, Roche Diagnostics GmbH, Mannheim, Germany). Microelectrodes were attached at the bottom of the wells for impedance-based detection of attachment, spreading and proliferation of the cells. Initially, 100 µL of cell-free growth medium (10% FBS) was added to the wells. After leaving the devices at room temperature for 30 min, the background impedance for each well was measured. Cells were harvested from exponential phase cultures by a standardized detachment procedure using 0.05% Trypsin-EDTA (Invitrogen NV/SA, Merelbeke, Belgium) and counted automatically with a Scepter 2.0 device (Merck Millipore SA/NV, Overijse, Belgium), Fifty µL of the cell suspension was seeded into the wells (20, 40, 80, 100, 200, 400 and 800 cells/well for proliferation, 1000 cells/well for cytotoxicity experiments). The cell concentrations of 20, 100, 200 and 400 cells/well were considered for correlation with the SRB method described below. After leaving the plates at room temperature for 30 min to allow cell attachment, in accordance with the manufacturer's guidelines, they were locked in the RTCA DP device in the incubator and the impedance value of each well was automatically monitored by the *xCELLigence* system and expressed as a *Cell Index* value (CI). Water was added to the space surrounding the wells of the E-plate to avoid interference from evaporation. For proliferation assays, the cells were incubated during ten days in growth medium (10% FBS) and CI was monitored every 15 min during the first six hours, and every hour for the rest of the period. Two replicates of each cell concentration were used in each test. For cytotoxicity experiments, CI of each well was automatically monitored with the *xCELLigence* system every 15 min during the overnight recovery period. Twenty-four hours after cell seeding, cells were treated during a period of 72 hours with paclitaxel (0, 1, 2, 5, 10, 20, 50 and 100 nM) dissolved in phosphate buffered saline (PBS). PBS alone was added to control wells. Each concentration was tested in duplicate within the same experiment. CI was monitored every 15 min during the experiment. Three days after the start of treatment with paclitaxel, CI measurement was ended.

### xCELLigence Real-Time Cell Analysis (RTCA): migration and invasion

Cell migration and invasion experiments were performed using modified 16-well plates (CIM-16, Roche Diagnostics GmbH, Mannheim, Germany) with each well consisting of an upper and a lower chamber separated by a microporous membrane containing randomly distributed 8 µm-pores. This setup corresponds to conventional Transwell plates with microelectrodes attached to the underside of the membrane for impedance-based detection of migrated cells. Prior to each experiment, cells were deprived of FBS during 24 hours. Initially, 160 µL and 30 µL of media was added to the lower and upper chambers respectively and the CIM-16 plate was locked in the RTCA DP device at 37°C and 5% CO_2_ during 60 minutes to obtain equilibrium according to the manufacturer's guidelines. After this incubation period, a measurement step was performed as a background signal, generated by cell-free media. To initiate an experiment, cells were detached using TrypLE Express™ (Invitrogen, Merelbeke, Belgium), resuspended in serum-free (SF) medium, counted and seeded in the upper chamber applying 3×10^4^ cells in 100 µL. After cell addition, CIM-16 plates were incubated during 30 minutes at room temperature in the laminar flow hood to allow the cells to settle onto the membrane according to the manufacturer's guidelines. To prevent interference from evaporation during the experiments, SF medium was added to the entire empty space surrounding the wells on the CIM-16 plates. Lower chambers contained media with or without FBS in order to assess chemotactic migration when exposed to FBS and background migration to SF medium as a negative control accordingly. Signals representing net chemoattraction were obtained by subtracting background (SF) values from the positive control (medium containing FBS) signals. Each condition was performed in quadruplicate with a programmed signal detection schedule of each three minutes during the first 11 hours of incubation followed by each five minutes for three hours and finally each 15 minutes to 24 hours of incubation.

A protocol identical to the above migration experiments was followed for invasion experiments added with the application of a layer of Matrigel on the upper side of the membranes and dynamic process follow-up during 50 hours. Aliquoted Matrigel (Basement Membrane Matrix, growth factor reduced, BD Biosciences, Erembodegem, Belgium) was thawed overnight on ice and mixed with ice cold SF medium to obtain two dilutions corresponding with ±190 µg/µL (10%, v/v) and ±63 µg/µL (3.3%, v/v) (Table 1). All Matrigel handling materials as well as the sealed packs containing CIM-16 upper chambers were stored ice cold overnight. Establishment of a Matrigel layer on the CIM-16 upper chamber membranes was achieved by adding 50 µL of the dilution sequentially on top of four membranes followed by removal of 30 µL, leaving a total of 20 µL Matrigel dilution. Subsequently, the coated upper chambers were incubated at 37°C to homogenously gelify during a minimum of four hours, followed by addition of 160 µL media to the lower and 30 µL SF media to the upper chambers. Equilibration and cell addition was carried out as described above.

**Figure 7:**
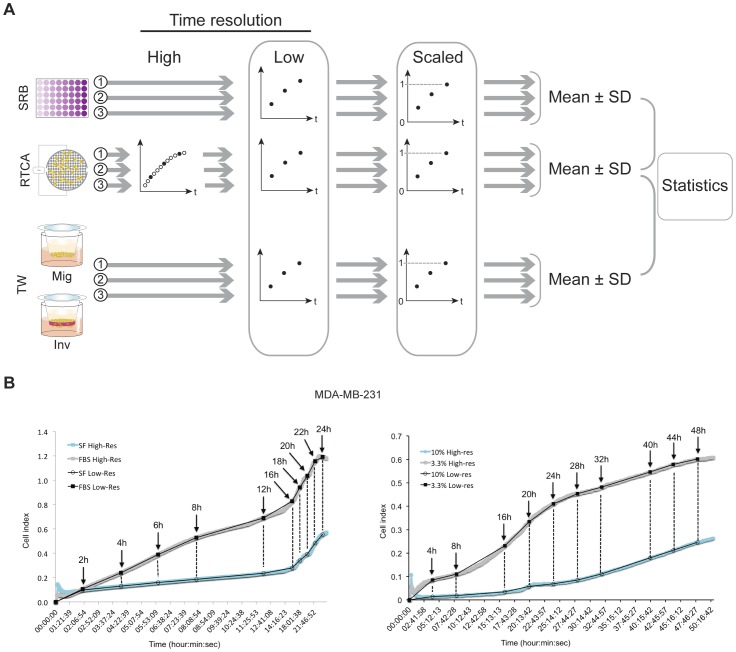
Prestatistical data processing. A. Schematic depiction of processing kinetic data generated by SRB, *xCELLigence* and Transwells. Raw data with high time resolution (*filled* and *empty circles*), resulting from independent *xCELLigence* experiments (1, 2, 3 and *grey arrows*) are reduced to a lower time resolution by selecting only the data points corresponding with the time points of endpoint detection (*filled circles* only). Subsequently, data have been normalized by dividing all values by the highest value recorded over all experiments per method, resulting in a modified Y-axis scale that ranges from 0 to 1. Finally, the normalized data have been averaged with calculation of SD for the three independent experiments per method. B. Reduction of high-resolution data, generated by *xCELLigence*, to a low resolution comparable with data from conventional assays. The example shows migration (left) and invasion (*right*) of MDA-MB-231 cells through two densities of Matrigel. The ten time points in the Transwell method (*black arrows*) were selected from the *xCELLigence* plots (*grey* and *blue*) to reconstruct a low-resolution graph (*black*), directly comparable to the Transwell data. An identical approach was applied for all other processes studied.

All data have been recorded by the supplied RTCA software (vs. 1.2.1). As described below in the “Prestatistical data processing and statistical analysis” section, original high-resolution data sets generated by *xCELLigence* were exported to MS Excel and reconstructed at a lower resolution by selecting only the data points corresponding with the respective time points of signal detection by the endpoint methods (Fig. 7). CI-data from cytotoxicity experiments have been normalized using the RTCA software to the last data point prior to treatment start. All other results (proliferation, migration and invasion) are based on raw data without CI-normalization and were processed as described above for comparison with conventional methodology.

### SRB assay: proliferation

Cells were harvested from exponential phase cultures by trypsinization, counted and plated in 48-well plates. To determine a proliferation curve and calculation of the doubling time, seeding densities ranged from 100 to 2000 A549 or MDA-MB-231 cells/well. Each concentration was tested six times within the same experiment. General cell culture conditions and culture medium used for this method were similar to those applied for the x*CELLigence* counterpart experiments, as well as applied cell densities (100, 500, 1000 and 2000 cells/cm^2^). Every day, one plate was fixed by the first step of the SRB assay: culture medium was aspirated prior to fixation of the cells by addition of 200 µl cold 10% trichloroacetic acid. After one hour incubation at 4°C, cells were washed five times with deionized water and left to dry. After collection of all plates during ten days, the following steps of the SRB test were performed as described previously [13], [14]. Shortly, the cells were stained with 200 µl 0.1% SRB dissolved in 1% acetic acid for at least 15 minutes and subsequently washed four times with 1% acetic acid to remove unbound stain. The plates were left to dry at room temperature and bound protein stain was solubilized with 200 µl 10 mM unbuffered TRIS base (tris(hydroxymethyl)aminomethane) and transferred to 96 wells plates for optical density reading at 540 nm (Biorad 550 microplate reader, Nazareth, Belgium). Cell doubling time was calculated from the exponential phase of the growth curve.

### SRB assay: cytotoxicity

Cells were harvested as described above. In order to assure exponential growth during the experiments, seeding density was 10^3^ A549 cells per well and 10^3^ MDA-MB-231 cells/well. After an overnight recovery period, treatment with paclitaxel (0–100 nM) dissolved in PBS was started. Control wells were added with PBS. Each concentration was tested six times within the same experiment. After 72 hours incubation with paclitaxel, survival was determined by the SRB assay as described above. IC_50_ values, representing the drug concentration causing 50% growth inhibition, were calculated using WinNonlin software (Pharsight, Mountain View, USA).

### Transwell migration assay

Comparative migration experiments were conducted using a conventional 24-well Transwell system (6.5 mm Transwell® (#3422), Corning®, NY, USA) with each well separated by a microporous polycarbonate membrane (10 µm thickness, 8 µm pores) into an upper (“*insert*”) and a lower chamber (“*well*”). After 24 hours of serum deprivation, cells were detached using TrypLE™ Express (Invitrogen, Merelbeke, Belgium), counted and resuspended in media without FBS to obtain equal cell densities (2.1×10^5^ cells/cm^2^) as applied in the *xCELLigence* RTCA DP system with respect to the membrane seeding surface of both techniques (classic Transwell® membrane surface 0.33 cm^2^, RTCA DP 0.143 cm^2^). A volume of 250 µL containing 7×10^4^ cells was plated to each insert and 600 µL medium was added to the wells. For each experiment, both chemotactic migration to medium containing 10% FBS and random migration with SF medium on both sides of the membrane have been assessed in parallel Transwell plates. At ten predetermined time points after incubation start (Fig. 3B), inserts were fixed and stained in duplicates and migration was quantitated using two commonly used methods. At each time point, cells were fixed and stained in a 20% methanol/0.1% crystal violet solution during three minutes at room temperature, followed by washing in deionized water to remove redundant staining [15]. Non-migrated cells remaining at the upper side of the membranes were carefully removed with cotton swabs and inserts were dried in darkness overnight. As fixing and staining was performed per set of two inserts with the remaining inserts to be further incubated until the following time point, inserts to be fixed and stained at a time point were transferred to a companion 24-well plate and the remaining inserts immediately replaced in the incubator. Using this approach unfavorable influences caused by continuous switching incubating cells between 37°C and ambient temperatures could be avoided. The following day stained membranes were pictured in three random non-overlapping fields at 10× objective and 10× eyepiece on a transmitted-light microscope (Leica DMBR, Leica Microsystems GmbH, Wetzlar, Germany) equipped with an AxioCam HRc camera (Carl Zeiss MicroImaging GmbH, Jena, Germany). A first method of quantitation was performed by processing all obtained images using ImageJ software (http://rsbweb.nih.gov/ij/). Each image (Fig. 4C, left) was color thresholded to obtain a binary (black & white – 8 bit) image with cellular material portrayed as saturated black areas (Fig. 4C, middle). As a next step all non-cellular artifacts, predominantly visible shadows of empty pores and debris, were removed from each image by performing the particle analysis function with a mask excluding all particles smaller than 100 to 250 pixels dependent on the experiment (Fig. 4C, right). Masking thresholds were set by comparing binary images with their original phase contrast counterparts [16]. Degree of migration for each time point per experiment was determined by calculating the average pixel area of the three fields in duplicate. Inserts were subsequently submerged in 300 µL 1% SDS/1× PBS in order to lyse migrated cells and extract crystal violet stain [17]. Submerged inserts were incubated in darkness overnight on a plate shaker at medium speed to ensure complete lysis. The following day 200 µL of each lysate was transferred to a 96-well plate for optical density (OD) measurement at 590 nm using a Powerwave X microplate scanning spectrophotometer (Bio-Tek, Bad Friedrichshall, Germany), representing a second cell migration quantitation method. Cell-free inserts containing only medium had been included in duplicate throughout each experiment as OD background controls. Reported OD data represent average background-corrected values ± SD obtained from three independent experiments in duplicate.

### Transwell Matrigel Invasion assay

Reference cell invasion experiments were carried out using a Transwell plate system as described for migration experiments, added with the application of Matrigel as extracellular matrix component. Matrigel dilutions were prepared as described above for the *xCELLigence* RTCA invasion assay. In order to obtain Matrigel surface area densities synchronized with the CIM-16 plates used for *xCELLigence*, two dilutions of 20% and 7.7% (v/v) have been prepared in ice cold SF medium corresponding with ±190 µg/µL and ±63 µg/µL respectively, as applied in a volume of 20 µL per insert membrane, identical to the CIM-16 upper chamber coating volume (Table 1). All other conditions regarding culturing, cell seeding density and serum deprivation were identical to the Transwell migration assays described above. At a panel of ten predetermined time points, inserts were fixed and stained in duplicates and invasion was quantified by OD reading at 590 nm after overnight extraction of the crystal violet stain. Reported OD data represent average background-corrected values ± SD obtained from three independent experiments in duplicate.

### Prestatistical data processing and statistical analysis

All data recorded using the *xCELLigence* RTCA system have been processed using MS Excel in order to obtain data series with the same resolution as the data recorded by the conventional reference methods (SRB, Transwell). This has been performed by selecting only the *xCELLigence*-generated values corresponding to the time points that have been used in the reference methods, thus leading to a reconstruction of the studied process dynamics at a lower time resolution (Fig. 7A, B).

Furthermore, to eliminate differences in units of measurement between the compared methods (*xCELLigence* CI, OD, pixel area), all data have been reduced to a (0–1) scale. This was done by considering all data gathered over three performed experiments per method and subsequently dividing all data by the single maximal value obtained, thus reducing this value to one (Fig. 4B and 7A). As these interventions do not influence proportionality nor variance levels of the data, comparable series of dynamically generated results were obtained for further statistical processing. For cell migration experiments, random migration signals (SF) were subtracted from the chemotactic migration signals (FBS) per time point to generate dynamic profiles of net chemoattraction (Fig. 4B).

All statistical analyses were performed using the statistical package R, version 2.13.1 (www.r-project.org). Correlations were calculated according to the Spearman's rank correlation method. Intra- and inter-experimental variances were assessed for each quantitation method separately using a mixed model approach with time as fixed and biological replicate as random effect. The standard deviation of the random intercept (inter-experimental variance) as well as the residual standard deviation (intra-experimental variance) was obtained through a variance component analysis. Due to differences in these values regarding the cell migration data, calculations were split up into “early” (before ten hours incubation) and “late” (after ten hours incubation) measurements. All cell migration data analyses were performed on background (SF)-reduced signals representing pure chemoattraction. Comparison of background migratory (negative control – SF) signal detection between the three quantitation techniques (pixel area calculation, OD and RTCA CI) was performed by fitting a mixed linear model where the signal was regressed on time, technique and their interaction. Biological replicates were entered as a random effect. A likelihood ratio test was performed to test the significance, expressed as a p-value, of the interaction term time-technique.

## Supporting Information

Table S1 **Variance component analysis of proliferation, cytotoxicity, migration and invasion.** All values expressed as the square root of the variance (σ^2^). σ_b_ Variance between independent experiments (“between”). σ_w_ Variance within one experiment (“within”). Low ±10^2^ and ±5×10^2^ cells/cm^2^. High ±10^3^ and ±2×10^3^ cells/cm^2^. Early Before 10 hours incubation. Late After 10 hours incubation. ND Not done. * Matrigel dilution in SF medium (v/v).
(DOCX)
