# Supplementary material for: Comparative Analysis of Dynamic Cell Viability, Migration and Invasion Assessments by Novel Real-Time Technology and Classic Endpoint Assays
Source: PLoS One. 2012 Oct 19;7(10):e46536. doi: 10.1371/journal.pone.0046536 (PMC3477108; doi:10.1371/journal.pone.0046536)
Supplement: Table S1 — Variance component analysis of proliferation, cytotoxicity, migration and invasion. All values expressed as the square root of the variance (σ2). σb Variance between independent experiments (“between”). σw Variance within one experiment (“within”). Low ±102 and ±5×10^2^ cells/cm2. High ±103 and ±2×10^3^ cells/cm2. Early Before 10 hours incubation. Late After 10 hours incubation. ND Not done. * Matrigel dilution in SF medium (v/v). (DOCX) [file pone.0046536.s001.docx]

**Supporting Information**

**Table S1.** Variance component analysis of proliferation, cytotoxicity, migration and invasion.

|  |  | |  | **MDA-MB-231** | | **A549** | |
| --- | --- | --- | --- | --- | --- | --- | --- |
|  | **Quantitation Method** | |  | **σ_b_** | **σ_w_** | **σ_b_** | **σ_w_** |
| **Proliferation** | |  |  |  |  |  |  |
|  | **SRB** | | **Low** | 0.01521 | 0.04793 | 0.04196 | 0.07290 |
|  |  | | **High** | 0.00163 | 0.04379 | 0.03392 | 0.06978 |
|  | **xCELLigence** | | **Low** | 0.01221 | 0.05249 | 0.01247 | 0.02824 |
|  |  | | **High** | 0.02051 | 0.08182 | 0.04321 | 0.05760 |
|  |  | |  |  |  |  |  |
| **Cytotoxicity** |  | |  |  |  |  |  |
|  | **SRB** | |  | 0.02398 | 0.02026 | 0.03870 | 0.03536 |
|  | **xCELLigence** | |  | 0.03440 | 0.04258 | 0.02264 | 0.03820 |
|  |  | |  |  |  |  |  |
| **Migration** |  | |  |  |  |  |  |
|  | **Pixel area** | | **Early** | 0.11181 | 0.09805 | 0.06954 | 0.07600 |
|  |  | | **Late** | 0.11619 | 0.07709 | 0.03147 | 0.02147 |
|  | **OD** | | **Early** | 0.04282 | 0.06929 | 0.02862 | 0.03437 |
|  |  | | **Late** | 0.12913 | 0.04380 | 0.04435 | 0.05112 |
|  | **xCELLigence** | | **Early** | 0.08794 | 0.07811 | 0.03793 | 0.02898 |
|  |  | | **Late** | 0.15776 | 0.08250 | 0.26603 | 0.07069 |
|  |  | |  |  |  |  |  |
| **Invasion** |  | |  |  |  |  |  |
|  | **OD** | | **20%*** | 3.00E-11 | 0.09 | ND | ND |
|  |  | | **7.7%*** | 0.05 | 0.05 | ND | ND |
|  | **xCELLigence** | | **10%*** | 0.06 | 0.1 | ND | ND |
|  |  | | **3.3%*** | 0.13 | 0.07 | ND | ND |
|  |  | |  |  |  |  |  |
